# Comparison of canine stifle kinematic analysis after two types of total knee arthroplasty: A cadaveric study

**DOI:** 10.14202/vetworld.2020.956-962

**Published:** 2020-05-20

**Authors:** Chaiyakorn Thitiyanaporn, Nattapon Chantarapanich, Somchai Sompaisarnsilp, Naris Thengchaisri

**Affiliations:** 1 Department of Companion Animal Clinical Sciences, Faculty of Veterinary Medicine, Kasetsart University, Bangkok, 10900, Thailand; 2 Department of Mechanical Engineering, Faculty of Engineering at Sriracha, Kasetsart University, Chonburi, 20230, Thailand; 3 Faculty of Veterinary Medicine, Rajamangala University of Technology Tawan-ok, Chonburi, 20110, Thailand

**Keywords:** cranial cruciate ligament, dogs, osteoarthritis, total knee arthroplasty

## Abstract

**Background and Aim:**

Osteoarthritis is a common consequence of cranial cruciate ligament rupture (CCLR) in the canine stifle. Total knee replacement is a valuable method for managing end-stage osteoarthritis.

**Materials and Methods:**

Two new designs of total knee replacement implants were fabricated with information from computed tomography scans. Canine hind limbs of cadavers were tested with a biomechanical testing machine with C-arm fluoroscopy. The four groups tested were as follows: Intact stifles (INTACT), CCLR, total knee arthroplasty (TKA) with a peg on top of the tibial component (TKAP), and TKA with no peg on top of the tibial component (TKAN). Extension, flexion, adduction, abduction, internal rotation, external rotation, cranial translation, caudal translation, and range of motion were measured.

**Results:**

The cranial translation of the tibia relative to the femur increased significantly after cutting off the cranial cruciate ligament. After arthroplasty, adduction/abduction and cranial/caudal translation within the TKAN group was increased compared with the intact stifle group. In the TKAP group, only adduction was greater than it was in the intact stifle group.

**Conclusion:**

The design of the prosthesis used for the TKAP group was more appropriate for total knee replacement in dogs than the design of the prosthesis for the TKAN group.

## Introduction

Osteoarthritis causes orthopedic pain, decreased activity, and lameness in dogs and cats [1-4]. The treatment options for severe osteoarthritis are limited to medical management, arthrodesis, or amputation [5]. The most common cause of stifle osteoarthritis is injury to the cranial cruciate ligament [6,7]. At present, many treatment options for cranial cruciate ligament injury are available. Although the ligament rupture stifle can be corrected by surgery, severe osteoarthritis is evidence of cranial cruciate ligament rupture (CCLR). The loss of the cranial cruciate ligament results in excessive motion of the joint, leading to higher pressure due to the force on the medial compartment, which rubs the articular cartilage and the medial meniscus. In engineering terms, the higher pressure acting on the surface causes wear during articulation. Over time, the cartilage and meniscus progressively deteriorate, gradually tearing the layer of extracellular matrix and eventually leading to bone-on-bone contact. Nowadays, tibial plateau leveling osteotomy (TPLO) is a standard surgical technique for the treatment of CCLR. This procedure changes the tibial plateau slope and increases stifle joint stability. However, osteoarthritis is still a consequential effect. In severe states of osteoarthritis, TPLO cannot cope efficiently with the disease.

Total knee arthroplasty (TKA) is considered a better option than TPLO. It has been used to treat severe osteoarthritis in dogs over the past 20 years [5]. TKA is an operation to replace degenerative articulated surfaces with an artificial prosthesis to restore motion in the knee. In the TKA prosthesis for dogs, the femur compartment is metallic and the tibial compartment is polymeric. Both cement and cement-less designs of the TKA prosthesis for dogs are commercially available [8]. The ideal TKA prosthesis should mimic the anatomy and motion of a normal knee joint [5]. However, the varying conditions of the stifle among different types of dogs complicate the use of a commercial TKA prosthesis for treatment. With the diversity of dog breeds, it is difficult for a standard prosthesis to fit and mimic the joint anatomy of any one specific breed. In light of these complications, custom-made total knee replacement components for dogs are available and fit well to the problem knee joint [9].

At present, two commercial total knee replacement products are available: Canine Total Knee (BioMedtrix, Boonton, NJ, USA) and GenuSys Knee (Innoplant, Medizintechnik, Hannover, Germany). Although commercial TKA prostheses are available, few studies have analyzed kinematics after total knee replacement [10,11]. For custom-made total knee replacement prostheses, there are several types of designs and prosthesis systems. This study used custom-made total knee replacement systems with one type of femoral component and two types of tibial components, including one with and one without a peg on top of the tibial component.

The main purpose of this study was to compare kinematic changes following two types of total knee replacement, CCLR stifle, and intact stifle. The hypothesis was that there would be no significant difference in kinematics between the CCLR stifle and the intact stifle or between the two types of prostheses.

## Materials and Methods

### Ethical approval

All limb samples of this study were collected from cadaveric dogs. They were died with other causes that unrelated to the experiment. The ethical approval is not necessary.

### Knee prosthesis design and manufacturing

A knee prosthesis consists of two parts: A femoral component and a tibial component. The design of the knee prosthesis is customized based on the anatomical geometry of the knee. Canines were scanned using computed tomography (CT) data, which produced a stack of two-dimensional digital imaging and communications in medicine images. These images were then used to construct the three-dimensional (3D) models of the knee by thresholding the proper Hounsfield unit of bone. The 3D models included the distal femur, the proximal tibia, and the proximal fibula exported into polygon form.

To design the femoral component, the curves of the femoral condyle, the patella trochlear, and the cross-sectional geometry perpendicular to these curves were extracted from the 3D femur models. These curves were used to create the articulated surface of the femoral component. The inner surface of the femoral component, attached to the bone, was divided into five surfaces, comprising the anterior plane, the posterior plane, the distal plane, and two chamfer planes between them. The two-peg type was designed as extrusion part from the distal plane. The pegs are intended to intrude into cancellous bone for increased rotation stability.

For the tibial component, the dimensions were based on the subchondral cross-section shape of the proximal tibia. It was 7 mm below the plateau surface. The shape of the subchondral cross-section was approximated into a trapezoid shape. The height of the trapezoid was obtained from the distance of the cross-section in the cranial-caudal (CC) direction. The width of the trapezoid was derived from distances in the medial-lateral (ML) direction. The narrower width was obtained from the ML direction at 2/5 of the CC direction, whereas the wider width was obtained from the ML direction at 4/5 of the CC direction. The articulated surface of the tibial tray was designed to match up with those knee component curves. The inner surface of the tibial tray attached to the bone was planar. The fin was also attached to the inner surface. It is designed to insert into cancellous bone to increase rotational stability. There were two designs of the upper surface. One design had one peg located at the middle of the upper surface. The peg is intended to prevent excessive CC translation and over-hyperextension. The second design did not include a peg.

**Figure-1:**
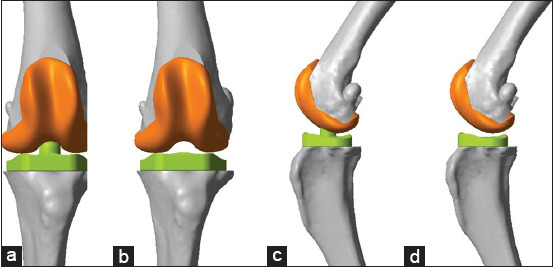
Prosthesis from the front view and lateral view. (a) Front view of implant on knee with peg on top of tibial implant (TKAP group), (b) front view of implant on knee with no peg on top of tibial implant (TKAN group), (c) lateral view of implant on knee with peg on top of tibial implant (TKAP group), (d) lateral view of implant on knee with no peg on top of tibial implant (TKAN group).

**Figure-2:**
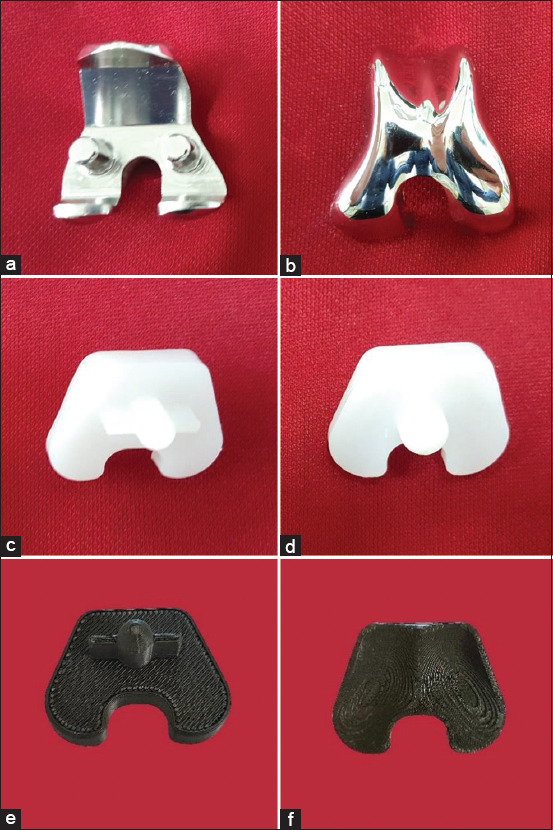
The femoral component and the tibial component of the prosthesis. (a) Bone contact surface with two pegs on the femoral component, (b) top surface of the femoral component, (c) bone contact surface with peg and fin (TKAP group), (d) top surface of the tibial component with peg (TKAP group), (e) bone contact surface with peg and fin (TKAN group), (f) top surface of tibial component with no peg (TKAN group).

Both components were designed using advanced computer-aided design (CAD) software that allows for the creation of complex surfaces. Before manufacturing, the design was validated and verified with a veterinarian using 3D printed models (Figure-1). After validation and verification, the 3D CAD design in parametric format was transferred to computer-aided manufacturing software to generate the tool paths for computer numerical control machine manufacturing. Knee components were made of cobalt chromium (CoCr), and tibial components were made of ultra-high molecular weight polyethylene (UHMWPE). CoCr is a metallic biomaterial, and its properties are good for wear resistance during knee joint motion. UHMWPE is a polymeric biomaterial, and its properties are good for energy absorption (Figure-2).

After manufacturing, the knee components and tibial trays were cleaned to remove debris left from the machining processing. They were packed in a plastic tray and transferred to a biomechanical testing laboratory at the Faculty of Veterinary Medicine, Kasetsart University.

### Customized osteotomy guide design

An osteotomy guide is a surgical instrument for cutting bones to prepare the prosthesis attachment site. A knee osteotomy guide was designed to place a prosthesis on the femoral condyle, whereas a tibial osteotomy guide was designed to place a prosthesis on the tibial plateau.

To develop the osteotomy guide, it was necessary to perform pre-operative planning by locating the proper position of the prosthesis at the joint according to the surgical procedure. After that, the osteotomy guide was created. The outer surface of the osteotomy guide was planar, whereas the inner surface conformed to the bone shape.

For the knee osteotomy guide, five inner planes of knee prosthesis were used to create the slot for the oscillating saw. The width of the slots was larger than the thickness of the saw blades, and the length of the slots was sufficient for cutting through the bone. Two Ø1.8 mm Kirschner wire holes were added to secure the osteotomy guide to the bone during the osteotomy procedure. The procedure for the tibial guide design was similar to that used for the knee osteotomy guide, but only one plane determining the bone removal thickness was included. An additional one Ø1.8 mm Kirschner wire hole was used to secure the osteotomy guide.

**Figure-3:**
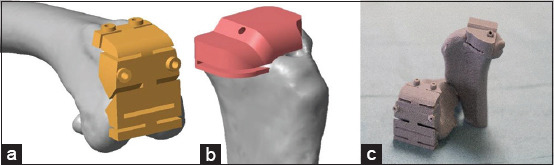
Osteotomy guide. (a) distal femur part, (b) proximal tibia part, (c) plastic osteotomy guide on the plastic bone model.

Both osteotomy guides were designed using CAD software. All 3D models were exported in a stereolithography (STL) file format, which can be used for 3D printing machines (M200, Zortrax SA, Poland). The STL files were sliced into layers and generated the support for printing. The key printing parameters were 0.09 mm layer thickness with a 100% dense internal structure. Acrylonitrile butadiene styrene (ABS) was selected as the printing material. ABS is biocompatible and widely used for temporary bone contact instruments. After the printing was finished, the guides were removed and cleaned (Figure-3).

### Limb samples

Six left hind limbs were collected from the cadavers of dogs that died from unrelated experimental reasons. The left hind limbs were cut in the proximal femur and distal tibia areas after weighing the cadavers. Their average body weight was 35.9±9.93 (mean±SD) kg. The average body condition score was 3.8±0.76/5. The average age was 8.8±1.10 years and 3.2±2.59 months. The hind limb cadavers were kept in –20°C until the experiment was conducted. Hind limbs were thawed in 4°C 24 h before the experiment.

The proximal part of the femur and the distal part of the tibia were fixed with a special design cup made of polymethyl methacrylate.

### Surgical procedure

**Figure-4:**
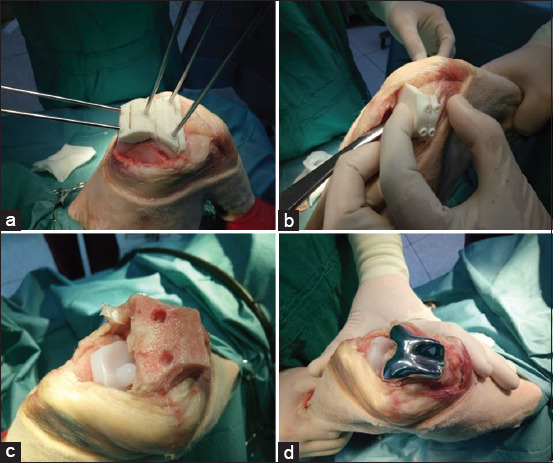
Steps of the total knee arthroplasty procedure. (a) The femur osteotomy guide was fixed to the distal femoral condyle with 1.8 mm Kirschner wires, (b) the tibial osteotomy guide was placed on the top of the proximal tibia before being fixed with Kirschner wire, (c) the ultra-high molecular weight polyethylene tibial component with the peg was placed on top of the proximal tibia and fixed with bone cement. The distal femur was cut and drilled to prepare it for the cobalt chromium femur component. (d) The distal part of the femur component was placed on the distal femur and fixed with bone cement.

Lateral arthrotomy was performed. The meniscus, cranial cruciate ligament, and caudal cruciate ligament in the stifle joint were removed. The femur osteotomy guide was fixed to the distal femoral condyle with a 1.8 mm diameter Kirschner wire. The distal femoral condyle was cut following the template pattern with an oscillating saw. Two 8 mm holes were drilled in the distal surface of the femur to fix the prosthesis with bone cement. The bone-cutting template of the proximal tibia was fixed to the proximal tibia with a 1.8 mm Kirschner wire. The lateral and medial collateral ligaments of the stifle joint were protected by gauze. The proximal tibia was cut with an oscillating saw following the bone cutting template. An 8 mm hole was drilled in the center of the cut proximal tibia area for inserting the stem of the tibial implant. The bone cement was applied to the proximal tibia cut surface. The proximal tibia prosthesis was attached to the proximal tibia cut and left until the bone cement set. Bone cement was applied to the surface of femur cut. The distal femur prosthesis was implanted on the bone cement surface using the impaction method and left until the bone cement set (Figure-4). The joint capsule was sutured with nylon number 1. Subcutaneous and skin layers were sutured routinely.

### Mechanical testing procedure

**Figure-5:**
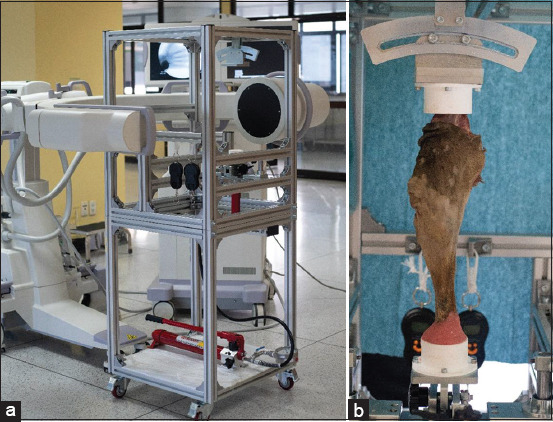
(a) Biomechanical testing machine with C-arm fluoroscopy, (b) limb cadaver fixed on the mechanical testing machine by acrylic and weight loaded on the limb sample.

The biomechanical testing machine was codesigned by Author #1 and Author #2. All testing machine parts were manufactured and assembled at the Faculty of Engineering at Sriracha, Kasetsart University, before the testing machine was transported to the Faculty of Veterinary Medicine, Kasetsart University, for the biomechanical testing procedure. The sample cup was fixed to the biomechanical testing machine by screws and weight loading under the fixed sample. Mechanical testing of the stifle was done using a joint biomechanical testing machine with C-arm fluoroscopy (SIROMOBIL Compact L, SIEMENS, Germany) (Figure-5). Intact stifles (INTACT) were tested for extension, flexion, internal rotation, external rotation, abduction, adduction, cranial translation, and caudal translation. The extension, flexion, internal rotation, external rotation, abduction, and adduction were measured in degrees. Cranial and caudal translation was measured in millimeters. After the tests on INTACT, the cranial cruciate ligament of the stifles was resected and tested using an identical procedure to that used on the INTACT. Then, TKA was performed on the stifles. Some prostheses used for TKA included a peg on the tibial component, and others included no peg. Radiographic pictures were collected and evaluated for deviation of the axis of the stifle joint using the image analysis module in CAD software (VISI, Vero, UK).

### Statistical analysis

The stifles were categorized into four groups: INTACT (intact stifle), CCLR, Total knee arthroplasty with a peg on top of the tibial implant (TKAP), and total knee arthroplasty with no peg on top of the tibial implant (TKAN). The range of motion in degrees of abduction, adduction, internal rotation, and external rotation and the range in millimeters of cranial translation and caudal translation in each group were analyzed with an analysis of variance. The means were compared with a Tukey-Kramer multiple comparison test. Significant different level was accepting with p<0.05.

## Results

Six stifles were tested for extension, flexion, abduction, adduction, internal rotation, external rotation, cranial translation, caudal translation, and range of motion in the INTACT, CCLR, TKAP, and TKAN groups. The extension and flexion values did not differ among the groups.

The abduction values in the INTACT, CCLR, and TKAP groups were lower than in the TKAN group, whereas the adduction values of TKAP and TKAN groups were higher than in the INTACT and CCLR groups. Internal and external rotation values of the groups did not differ (Table-1).

**Table-1:** Mean±SD in degrees of the extension, flexion, range of motion, abduction, adduction, internal rotation, and external rotation in the INTACT, CCLR, TKAP, and TKAN groups.

Parameters	INTACT	CCLR	TKAP	TKAN
Extension	145.22±11.92	148.46±12.04	137.84±14.32	138.63±15.18
Flexion	27.38±9.10	29.40±9.86	18.4±8.71	16.93±8.20
Range of motion	117.84±7.22	119.06±10.18	119.44±10.92	121.7±8.43
Abduction	4.52±1.73	5.28±1.92	4.50±1.61	10.50±1.73*
Adduction	6.38±2.64	9.18±2.64	18.4±5.65*	20.20±4.46*
Internal rotation	13.20±7.69	13.10±8.91	17.75±13.52	16.00±3.61
External rotation	10.40±5.55	10.20±6.22	6.80±4.09	6.17±7.65

* Within a row, mean significant difference (p<0.05). INTACT=Intact stifles, CCLR=Cranial cruciate ligament rupture, TKAP=Total knee arthroplasty with peg, TKAN= Total knee arthroplasty with no peg, SD=Standard deviation

The cranial translation values of the CCLR and TKAN groups were notably different from those of the INTACT and TKAP groups. The caudal translation values of the TKAN group were higher than in the INTACT, CCLR, and TKAP groups. The range of motion in all groups was not significantly different (Table-2).

**Table-2:** Mean±SD in millimeters of the cranial translation and caudal translation in INTACT, CCLR, TKAP, and TKAN groups.

Parameters	INTACT	CCLR	TKAP	TKAN
Cranial translation	1.51±0.80	4.87±1.68*	1.18±0.29	4.33±3.27*
Caudal translation	0.93±0.85	2.30±0.82	1.29±1.41	5.98±2.92*

* Within a frow, mean significantly difference (p<0.05). INTACT=Intact stifles, CCLR=Cranial cruciate ligament rupture, TKAP=Total knee arthroplasty with peg, TKAN= Total knee arthroplasty with no peg, SD=Standard deviation

## Discussion

The consequence after CCLR is osteoarthritis. This situation is the most common in dogs and cats. The management of osteoarthritis including non-surgical management and surgical management. For non-surgical management, the weight control, exercise and habitat management, nutritional supplement, non-steroid anti-inflammatory drug given, joint supportive brace, and other rehabilitation methods are recommended [12]. For surgical management, the total joint replacement is the most effective effect to the treatment of osteoarthritis in dogs and cats [13].

The first occurrence of TKA in dogs was published in human orthopedic research literature. The design of the femoral component was made from CoCr, and the tibial component was made of ultra-high molecular weight polyethylene [14]. The first commercial total knee replacement in dogs was implanted in a working dog with a comminuted fracture of the distal femur from a gunshot injury in 2005 [9]. Nowadays, both cement (BioMedtrix, NJ, USA) and cement-less (GenuSys Knee, Innovet, Hamburg, Germany), total knee replacements are available. However, custom-made total knee replacements are still necessary for dogs because commercial artificial knees may not fit the knee case of every dog breed.

In this study, both femoral component and tibial component prostheses were designed using the information from CT scan data of the left side stifle of a 25 kg Labrador Retriever. Although all cadaveric canines in this study were implanted with the custom-made prosthesis from the reference Labrador Retriever dog, the authors selected canines with bone and weight dimensions close to those of the reference dog to ensure the conformity of the prosthesis to all cadaveric canines. In addition, CAD pre-operative planning was used to adjust the attachment position of the prosthesis by aligning the prosthesis to the caudal surface and groove surface of the bone. Thus, the attachment position of the prosthesis in all cadaveric canines was optimal within small variations of bone dimensions. After CAD pre-operative planning, plane surfaces of the prosthesis were used in cutting the guide design to control the position during surgery.

The femoral component, the femoral trochlear, and both sites of the femoral condyle were designed based on the normal anatomy of the distal femur. On the bone contact surface, the authors in this study designed two pegs on the distal surface of the femoral component to prevent transverse rotation of the component, which subsequently increases stability. Two types of the tibial component were designed: One with a peg on the top surface and another without a peg on the top surface. In both types of tibial components, the bone contact surface included a fin. This fin can increase stability of the implant by preventing transverse rotation and increasing the fixation surface.

The advantage of the peg on top of the tibial component is that it can prevent the cranial translation of the tibia relative to the femur better than the non-peg type. In intact stifle joints, the tension of the cranial cruciate ligament limits the translation of the distal femur. Without the cranial cruciate ligament (or if loss of tension of the ligament occurs), the distal femur displaces more caudal. In general, in the TKAN prosthesis, without a peg on the top surface, there is no mechanical structure to limit such a caudal translation of the distal femur. With the peg-type tibial tray, the end slot at the intercondylar notch of the femoral component is in contact with the peg, limiting the CC translation, as shown in Table-2.

In addition, the peg limited the abduction-adduction. This can be explained by the fact that the peg is located at the center of the intercondylar slots of the femoral components, and the slot surface controls the rotational motion (abduction-adduction). The maximum possible abduction-adduction angles depend on the arctangent angle of height of the slot divided by the maximum gap between the peg and the slot surface. Thus, the thickness of the slot, the slot width, and the diameter of the peg play an important role in controlling abduction-adduction. If these parameters are designed inappropriately, joint stability cannot be achieved. The fit in the tolerance of the peg-slot may lead to stiffer motion, which hinders necessary motion of the mimicking intact joint, whereas loosening the tolerance may lead to instability of the joint. Without a peg on the top of the tibial component, the motion of the femur is not controlled. It rotates more easily compared with the peg type, as seen in Table-1, but abduction-adduction is limited by muscle and soft-tissue strength.

The only unfavorable performance of the peg type was in extension, because the peg hinders the motion when the peg is in contact with the slot. However, there was no significant difference between the peg type and the non-peg type, but a large difference compared with the intact stifle. This leads to higher load bearing around the caudal tibial plateau, which results in higher wear rate. Overall, the tibial component with the peg is preferable in design.

The cutting template used in this study was a custom-made ABS polymeric template. The cutting template was designed based on the CT scan information so that it would fit well to the femur condylar surface and the tibial plateau surface. To the best of the authors’ knowledge, this method of cutting is new for total knee replacement in canines because the commercial total knee replacement company provides a standard cutting template with the set of instruments. This ABS polymeric template makes a good cut and accurate chamfer that fit well to the bone contact surface of the femoral component and tibial components.

In this study, after cutting off the cranial cruciate ligament, cranial tibial translation increased by 3.36 mm compared with the intact stifle, whereas other parameters were not significantly different. A study by Arnocky *et al*. [15] found that cutting the cranial cruciate ligament resulted in a 150% increase in internal tibial rotation, an 8% increase in extension, and an increase in cranial translation of 9.5 mm in 15–20 kg dogs.

In a recent study, cutting the contact surface of the femoral and tibial medial and lateral collateral ligaments of the stifle played an important role in stifle stability. Both ligaments were preserved from trauma during an operation because the medial collateral ligament will be taught in both flexion and extension while the lateral collateral ligament will be taught in extension and relax in flexion. For this reason, the collateral ligaments controlled adduction, abduction, internal rotation, and external rotation in the extended stifle [16]. In the kinematic analysis of the knee joints in this study, the results of the TKAP group were similar to those of the intact group, except that adduction in the TKAP group was higher than normal. Yet the TKAN group had many parameters that differed from those in the intact stifle group, including abduction, adduction, cranial translation, and caudal translation. The TKAP group may have experienced better stifle stability than the TKAN group because the peg on the top surface of the tibial component of the implant can contribute to the stabilization of the stifle. However, in practice, the rehabilitation of muscles after TKA is very important, because the dogs with osteoarthritis always have problems with disuse atrophy of muscles. Muscle strength can provide stability and a well-functioning stifle after total knee replacement [17]. The difference in adduction in the TKAP group may not be affected.

This study used cadavers as test specimen. They provided sound results for the kinematic analysis of the stifle joint when it was intact, during CCLR, and after TKA. Nevertheless, the long-term use of the custom-made prosthesis could not be evaluated, especially the effect of physiological cyclic knee motion on the prosthesis. With the effect of load over time, the UHMPE tibial component, the peg and plateau surface in particular, can tear. Wear of the peg can increase extension, abduction, adduction, and CC translation. Wear of the plateau surface may lead to changes in internal and external rotation. The high impact on the peg due to extensive activity can accelerate wear and eventually cut the peg away. These long-term effects should be further investigated using *in vivo* or computer-aided engineering simulations.

## Conclusion

The design of total knee replacement components is important to making the knee return to normal function. A peg on top of the tibial component of a prosthesis can better control the cranial translation of the tibia relative to the femoral condyle, adduction, and abduction than a tibial component with no peg.

## Authors’ Contributions

CT gave the concept, designed the study, performed the experiment, performed statistical analysis, and wrote the manuscript. NC manufactured the mechanical testing machine, designed and fabricated the implants, performed the experiment, and data calculation, SS collected the samples and performed the experiment. NT designed the experiment and experimental suggestion. All authors contributed to drafting and revision of the manuscript. All authors read and approved the final manuscript.
